# Drosophila Answers to TDP-43 Proteinopathies

**DOI:** 10.1155/2012/356081

**Published:** 2012-04-18

**Authors:** Maurizio Romano, Fabian Feiguin, Emanuele Buratti

**Affiliations:** ^1^Department of Life Sciences, University of Trieste, Via A. Valerio 28, 34127 Trieste, Italy; ^2^International Centre for Genetic Engineering and Biotechnology, Padriciano 99, 34149 Trieste, Italy

## Abstract

Initially implicated in the pathogenesis of CFTR and HIV-1 transcription, nuclear factor TDP-43 was subsequently found to be involved in the origin and development of several neurodegenerative diseases. In 2006, in fact, it was reported for the first time the cytoplasmic accumulation of TDP-43 in ubiquitin-positive inclusions of ALS and FTLD patients, suggesting the presence of a shared underlying mechanism for these diseases. Today, different animal models of TDP-43 proteinopathies are available in rodents, nematodes, fishes, and flies. Although these models recapitulate several of the pathological features found in patients, the mechanisms underpinning the progressive neuronal loss observed in TDP-43 proteinopathies remain to be characterized. Compared to other models, Drosophila are appealing because they combine the presence of a sophisticated brain with the possibility to investigate quickly and massively phenotypic genetic modifiers as well as possible therapeutic strategies. At present, the development of TDP-43-related Drosophila models has further strengthened the hypothesis that both TDP-43 “loss-of-function” and “gain-of-function” mechanisms can contribute to disease. The aim of this paper is to describe and compare the results obtained in a series of transgenic and knockout flies, along with the information they have generated, towards a better understanding of the mechanisms underlying TDP-43 proteinopathies.

## 1. Introduction

Nowadays, Drosophila is one of the most widely used model organism for studying complex genetic and biological problems. Drosophila was introduced as an experimental model in the early twentieth century. Although there are several different Drosophila species which differ in habitat, morphology, and genetic background, *D. melanogaster* is the species with the highest degree of homology with humans. Over the years, many mutants of *Drosophila melanogaster* have been isolated and many transgenic strains have been created as model systems for the study of human disease [1], leading to the observation that more than 70% of human loci correlated with pathological conditions have orthologs in *Drosophila melanogaster* [2]. Compared to other animal models used to study the mechanisms underlying many disease onset/progression, Drosophila present several advantages: the life span is short (from 40 to 120 days) [3]; the genome is compact, with only 4 pairs of homologous chromosomes and roughly 13600 genes [4]. The importance of *Drosophila melanogaster* is further supported by the fact that it represents an animal model where the efficacy of putative therapeutic agents can be quickly screened and tested [1, 5]. Altogether, therefore, these characteristics make Drosophila an ideal model for gaining insights into the mechanisms by which genes cause disease in humans.

In particular, in the last ten years increasing attention has turned towards this animal in order to obtain a better understanding of neurodegenerative disorders [1, 5]. For this purpose, the most appealing feature of this organism consists in its high neuronal complexity, derived from an advanced brain capable of learning and memory, as well as from the presence of a nervous system made up of several specialized cytotypes belonging to ion channels, receptors, and neurotransmitters that are present also in humans. In conclusion, the degree of conservation between humans and flies has allowed to model consistently several human neurodegenerative disorders in the Drosophila brain [20].

 Among genes implicated in human disease, TDP-43 is a nuclear factor recently identified as a protein capable of playing a crucial role in the pathogenesis of FTLD, ALS, and other neurodegenerative diseases [21–24]. In ALS and FTLD patients, in fact, this protein (and its C-terminal fragments) has been found as the major components of preferentially cytoplasmic phosphorylated inclusions positive for ubiquitin and tau/*α*-synuclein negative [25, 26]. The mechanisms through which these inclusions may be connected with disease are still the subjects of debate [27].

 However, the fact that they may be acting as a cytoplasmic protein “sink” to trap the predominantly nuclear TDP-43 has suggested that the onset of neurodegenerative diseases might be the consequence of the progressive nuclear loss of function of TDP-43 trapped within the cytoplasmic inclusions (this does not rule out, of course, that various “gain-of-function” mechanisms may be playing a role in parallel) [22].

 In order to clarify these issues it is therefore important to find a suitable animal model that expresses a functional homologue of human TDP-43. In this respect, it is already known that the TDP-43 ortholog of *Drosophila melanogaster* (namely, TBPH) has the structure and in vitro functions similar to the human protein, at least for the repression of splicing of specific exons [36, 37]. Therefore, in consideration of this considerable homology between human and fruit fly TDP-43 orthologs there is a growing interest in utilizing Drosophilas as a model to characterize the functions of this protein. Here, we will review the various flies models that have been developed up to now to investigate the pathological hallmarks of the neurodegenerative diseases related to alterations of expression of wild-type or mutant TDP-43/TBPH.

## 2. Structural Divergences and Functional Conservation between TDP-43 and TBPH

**Figure 1:**
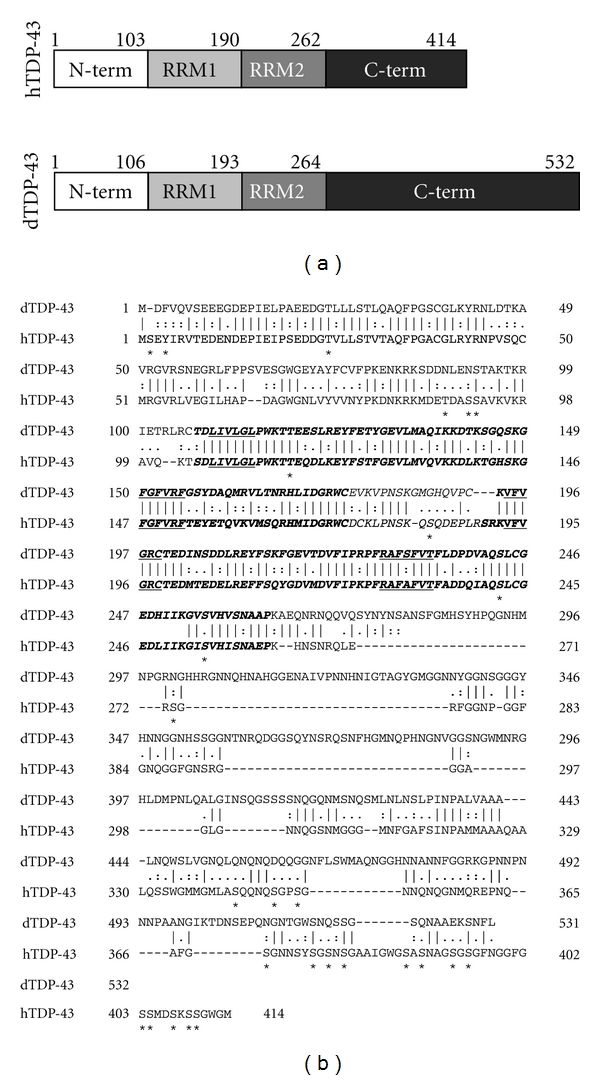
Comparison between human and fly TDP43. (a) Schematic representation of each domain (and amino acid position) in the two orthologs. (b) Alignment of the human (TDP-43) and fly (TBPH) proteins. The longest isoforms of human and fly proteins were used for this analysis (Uniprot accession no. Q13148 and O97468, resp.). Amino acids identical (∣) or similar (:) between the two proteins are indicated. Bold residues indicate the RNA recognition domains, RRM1 and RRM2. The RNP consensus sequences present in each RRM are highlighted (bold and underlined). All potential phosphorylation sites within human TDP-43 are indicated by asterisks. Importantly different studies have found a strong link between hyperphosphorylation at Serine379, Serine403, Serine404, Serine409, Serine410, and the inclusions of TDP-43 in FTLD-U/ALS [28–34].

 The TARDBP gene is a highly conserved gene throughout evolution and orthologs of human TDP-43 have been found in all higher eukaryotic species, including *Drosophila melanogaster*. In flies, the main homologue of human TDP-43 is a protein called TBPH (UniProt, O97468_DROME). The comparison of the longest protein isoforms shows that the region that includes two RNA recognition motifs (RRM1 and RRM2, Figure 1(a)) is more conserved than the C-terminal region (Figure 1(b)). In fact, pairwise amino acid alignment highlights the presence of 59% identity and 77% similarity between human and fruit fly N-terminals/RRMs, whereas the degree of identity and similarity of the C-terminus regions is only 18% and 22%, respectively.

 Notwithstanding these differences, many experiments have highlighted an extremely high degree of functional similarity and interchangeability between the human and fly proteins (see also below). First of all, both proteins share the same preference with regards to RNA binding specificities as recent studies have shown that TBPH is able to preferentially bind the same consensus (UG)n rich sequences tagged by the human ortholog [36]. Importantly, it has also been shown that TBPH has splicing inhibitory effects overlapping those of human TDP-43 both *in vitro* and *in vivo*. For example, *in vitro* assays have demonstrated that the Drosophila TDP-43 ortholog was able to interfere with CFTR exon splicing minigenes similarly to TDP-43 [36]. Even more strikingly, and similarly to what has been observed for the human protein [37, 38], the TBPH mutant without the C-terminal region lost this inhibitory activity. This observation has provided the first evidence that many, if not all, characterized functions of TDP-43, are common both for the human and fly protein and even extend to the same protein regions. In keeping with this view, subsequent *in vivo* studies have confirmed that C-terminal region of TBPH, despite its structural and sequence divergence from TDP-43, can also interact with the same nuclear partners of TDP-43 (i.e., hnRNPA2, hnRNPA1, hnRNPC, and hnRNPB1) [37].

 Altogether, therefore, these observations indicate that there is an extremely high degree of functional conservation through evolution from flies to human and this observation has represented the starting point for using Drosophila models to address the issue of TDP-43-dependant neurodegeneration.

## 3. TBPH Null Allele Drosophilas

Two complimentary approaches have been utilized for the genetic characterization of ALS-related pathways: loss and gain of function of the Drosophila TDP-43 ortholog and overexpression of TDP-43 variants presenting mutations found in ALS patients. Tables 1 and 2 summarize the TDP-43 knocked out and transgenic flies so far generated and the most relevant phenotypic features observed in these models.

## 4. Loss-of-Function Models

 With regards to loss of function, the general strategy has been to target disruption of the TBPH gene in Drosophila in order to analyze whether ALS-like phenotypes could be generated.

 The first TBPH-ko model used chromosomal deletions that removed the initial part of the gene regulatory region and the ORF [6]. Homozygous TBPH-ko flies were viable after embryogenesis and most of them were able to reach the pupal stage and undergo metamorphosis [6]. It was hypothesized that the progression through the first stages of developments is probably due to the permanence of residual gametic TBPH. However, a high percentage of TBPH-/-flies were unable to complete eclosion and remained trapped inside the pupal cages. Furthermore, TBPH-ko flies presented a dramatic decrease in flight and walking performances with spastic and uncoordinated movements [6]. As a result, the life span of these flies was significantly reduced. The analysis of presynaptic terminals at neuromuscular junctions (NMJs) highlighted that the TBPH-/-larvae show a reduction of axonal branches and synaptic boutons present inside the muscles [6].

 The second loss-of-function model was generated with a point mutation (G>A) that introduced a stop codon at residue 367 (Q367X) [7]. The semilethality of the homozygosity for TBPH deletion was confirmed by flies homozygous for the Q367X allele or flies treated with tubulin-Gal4-TBPH RNAi knockdown [7]. This study showed that TBPH can regulate dendritic branching in ddaE and ddaF neurons. In fact, whereas TBPH silencing (both by homozygosity for Q367X mutation or by whole-body RNAi) decreased dendritic branching, TBPH overexpression had the opposite results [7].

 Most importantly, both studies demonstrated that the functions of the human TDP-43 overlapped those of TBPH also *in vivo*. In fact, it was shown that overexpression of TDP-43 could efficiently rescue the phenotype caused by TBPH deficiency.

 A third null allele model of TBPH was generated by deleting the whole coding sequence of the TBPH gene [9]. Contrary to what was observed in previous reports, homozygous TBPH -/- flies died at the second instar larval stage. It should be noted, however, that this null allele model was used only to confirm *in vivo* the data obtained *in vitro* with human cell lines and no further molecular characterization was carried out. In particular, this model was used to confirm that the ability of TDP-43 to bind and regulate expression of the HDAC6 mRNA was conserved also by TBPH in Drosophila [9]. In conclusion, a proper comparison with the two previous models will have to await for a more extensive characterization of this model.

 Finally, a fourth null allele model of TBPH was created by deletion of 932 bp within the 5′ end of TBPH [17]. Similarly to the other models [6, 7], early lethal phenotype with alteration of locomotion was observed both in larvae and in the adult flies. On the other hand, the number of the synaptic boutons in the NMJ of these flies was increased (while it was decreased in the other models). The reasons for this discrepancy remain still unknown.

 Taken together, these studies suggest that the genetic background can significantly modulate or modify the effects of the genomic mutations. However, all these initial reports are strongly in agreement that depletion of TDP-43/TBPH can trigger neurodegeneration.

## 5. TDP-43 Transgenic Flies

 As intuitively obvious, a complimentary approach to the creation of TBPH-ko flies consists in the ubiquitous or tissue-specific overexpression and/or silencing of this factor by using the Gal4-UAS system. Consistently, different reports found that the expression of the wild-type form of TDP-43 (both TBPH and TDP-43) in the Drosophila eye causes a severe retinal degeneration with loss of cells [10–13, 18].

 In a likewise manner, expression of TDP-43 in mushroom bodies caused neuronal death and axonal loss [11] and overexpression of TDP-43 in motor neurons caused morphological and functional defects associated with cytoplasmic and axonal aggregation as well as axonal swelling [11]. Different TDP-43 toxicity has been also associated with its subcellular accumulation. For example, it was shown that cytoplasmic accumulation of TDP-43 is sufficient to induce degeneration in neuronal and nonneuronal tissues [12, 18]. Cytoplasmic inclusions of TDP-43 were toxic in eyes, muscles, and glia, whereas both cytoplasmic or nuclear TDP-43 accumulation were toxic in neurons of adult flies [18]. Finally, in some cases the ectopic expression of TDP-43 in eyes led to the detection of nuclear detergent-insoluble high-molecular-weight TDP-43 aggregates [11, 12]. Similarly to what is observed in mammalian systems [39, 40], TDP-43 toxicity was associated with increased levels of both nuclear and cytoplasmic TDP-43, independently from the presence of inclusions [18]. Indeed, a consistent observation in different reports was that the ectopic TDP-43 expression led to a dose- and age-dependent toxicity. In fact, the onset time and the severity of locomotion defects and neuronal loss were proportional to the levels of TDP-43 expression [8, 10, 11, 13, 18].

 Another distinguishing feature of TDP-43 proteinopathies is the presence of mutations occurring mainly within the C-terminal region of the TDP-43 protein (that have been identified mostly in both sporadic ALS and FALS patients [41]). Overexpression of artificial or disease-related TDP-43 mutants displayed variable levels of toxicity (see Table 1) probably depending on the exact level of overexpression with respect to wild-type protein. In fact, it is quite challenging to separate the eventual toxic effects of the mutation with respect to the general toxicity induced by TDP-43 overexpression. The expression of the ALS/FTLD-linked TDP-43 mutations A315T and M337V in flies resulted in an increased neurodegeneration (Table 2) [7, 8, 12, 13, 15], consistently with the phenotypes exhibited by the transgenic rodent models for the same mutations [39, 42, 43]. On the other hand, other disease-causing mutations expression in flies caused a variable increment of toxicity (Table 2) [7, 8, 13, 15].

 Other types of more artificial mutations, however, were found to be more consistent with regards to their effects. For example, this was the case for the impact of mutations within the nuclear localization and nuclear export sequences. In general, while the expression of the TDP-43 NLS-mutant in the fly eyes resulted in a stronger [12, 18] or comparable [8] degenerative phenotype compared to the effects of the TDP-43 wild-type expression, the TDP-43 NES-mutant was associated with a normal phenotype [12, 18].

 Finally, and most importantly, it is clear that the RNA-binding activity of TDP-43 is crucial for TDP-43 toxicity [8, 11, 19], whereas the C-terminal end seems to have milder impact on longevity, neuronal loss, and locomotion [8].

## 6. Genetic Interactions with TDP-43 in Drosophila

 A growing body of evidence indicates that TDP-43 abnormalities are not limited to ALS and FTLD but are present in many neurodegenerative and myodegenerative diseases [44–46]. Of course, several other genes have also been implicated in the pathogenesis of these diseases. Different studies have therefore addressed the problem of possible interactions between TDP-43 and some of these genes, in order to highlight the presence of common pathologic pathways or the existence of genetic modifiers of disease severity [41, 47–52].

**Figure 2:**
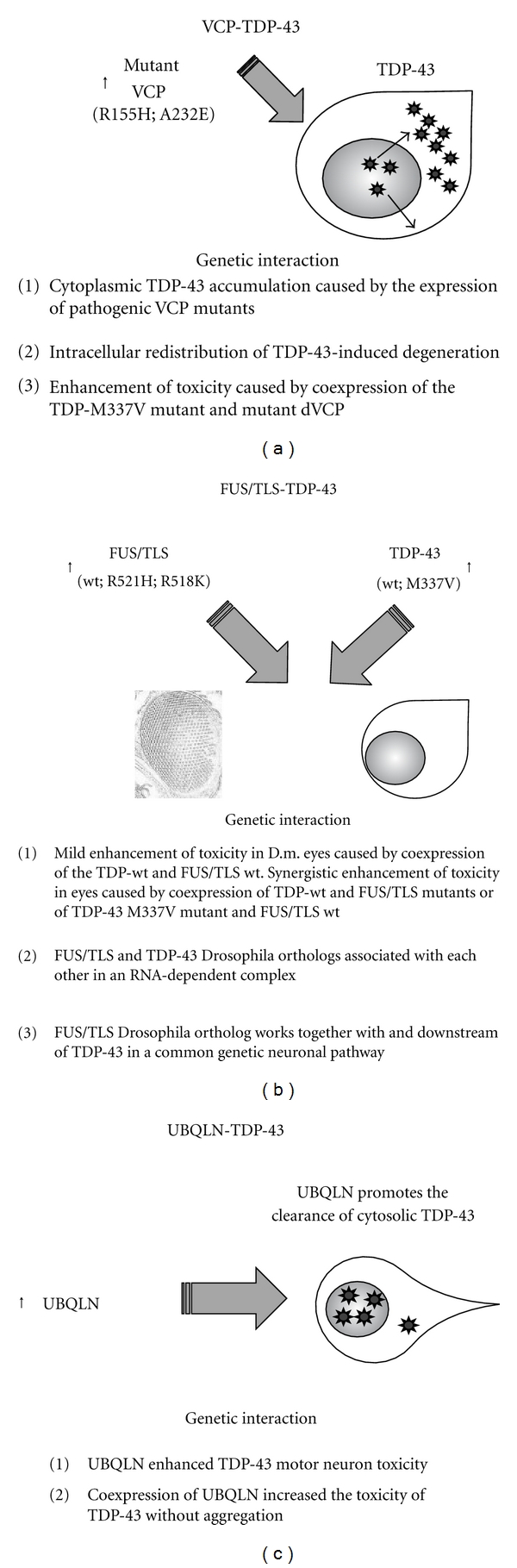
Genetic interactions of TDP-43/TBPH. (a) Schematic representation describing the effects of valosin-containing protein (VCP7) gene overexpression on TDP-43-toxicity in Drosophila melanogaster. (b) Schematic representation describing the effects of FUS/TLS gene overexpression on TDP-43-toxicity in Drosophila melanogaster. (c) Schematic representation describing the effects of UBQLN gene overexpression on TDP-43-toxicity in Drosophila melanogaster. The lines of evidence supporting the genetic interaction between the TDP-43 and these other genes are schematically summarized.

 The Valosin-Containing Protein (VCP) is a molecular chaperone VCP that segregates ubiquitinated substrates from multimeric protein complexes. Autosomal dominant mutations in the VCP gene have been identified in the Inclusion Body Myopathy associated with Paget's disease of bone and frontotemporal dementia (IBMPFD; MIM167320). Mutations in this gene exhibit variable penetrance and cause progressive muscular bony and neuronal degeneration with lethal exitus [53]. Most importantly, TDP-43 has been found within ubiquitinated cytoplasmic inclusions in the muscles of IBMPFD patients [44, 47]. In order to identify genes able to suppress neurodegeneration, a genetic screening was undertaken in a Drosophila model of IBMPFD created with disease-causing VCP mutations [12]. In this way, a genetic interaction between VCP and TDP-43 was highlighted (Figure 2(a)). In this model, it was observed that (1) the expression of pathogenic VCP mutants caused cytoplasmic TDP-43 accumulation *in vivo*; (2) the intracellular redistribution of TDP-43 was sufficient to induce degeneration; (3) enhancement of toxicity was obtained following coexpression of the TDP-M337V mutant with mutant dVCP [12]. These findings are consistent with a knockin IBMPFD mouse model that exhibited progressive cytoplasmic accumulation of TDP-43 and ubiquitin-positive inclusion bodies in quadriceps myofibrils and brain [48]. Thus, both animals apparently represent useful models for preclinical studies regarding this pathology.

 Another gene found mutated in familial and sporadic ALS is the one coding for fused in sarcoma/translocated-in-liposarcoma (FUS/TLS) protein [54, 55]. Similarly to TDP-43, FUS/TLS is a nuclear RNA-binding protein ubiquitously expressed [41]. Several ALS-causing mutations in this protein cause the redistribution of the protein to the cytoplasm and this accumulation leads to the formation of cytoplasmic inclusions in neurons and glial cells [54–56]. The coexistence of FUS- and TDP43-immunoreactive inclusions in an ALS patient carrying the TDP-43 G298S mutation suggested the FUS/TLS and TDP-43 might share some pathologic mechanisms leading to ALS [57]. Subsequent studies demonstrated that FUS/TLS and TDP-43 interact physically and functionally [41, 58, 59]. To better investigate this interaction, two Drosophila models of FUS/TLS-mediated ALS were obtained by generating a null allele of the fly FUS/TLS ortholog [60] and by expressing mutant human FUS/TLS in eyes and motor neurons [15]. In these models, the genetic interaction between FUS/TLS and TDP-43 was demonstrated through the worsening of neurodegenerative phenotypes following coexpression of wild-type or mutant FUS/TLS and wild-type or mutant TDP-43 [15, 60] (Figure 2(b)). Furthermore, it was confirmed that also the FUS and TDP-43 fly orthologs associate with each other in an RNA-dependent complex [60] as reported for the human proteins [50, 58, 61].

 Finally, recent studies have found that ubiquilin 1 (UBQLN) is another potential TDP-43 interactor [62]. UBQLN is a cytosolic protein involved in targeting misfolded proteins to the proteasome for degradation [63] and implicated in the pathogenesis of some neurodegenerative diseases [64, 65]. Experiments in mammalian cells showed that TDP-43 and UBQLN colocalize in cytosolic aggregates [62] and suggested that UBQLN might be involved in pathway controlling TDP-43 degradation. In order to investigate its genetic interaction with UBQLN, a Drosophila model of TDP-43 proteinopathy was generated (Figure 2(c)). Contrarily to expectations, however, coexpression of UBQLN increased the toxicity of TDP-43 without aggregation although the levels of TDP-43 were decreased and these results were further confirmed also in a mammalian cell line [10].

 Taken together, these observations provide evidence that many aspects of neurodegeneration are conserved from Drosophila to vertebrates and strengthen the utilization of the Drosophila models to study systematically modulators of TDP-43-induced neurodegeneration.

## 7. Genes and Proteins Regulated by TBPH in Drosophila

**Figure 3:**
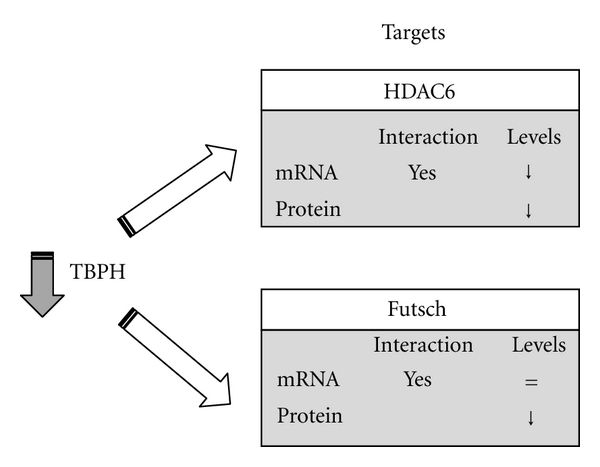
HDAC6 and futsch levels are controlled by TBPH in Drosophila. Two genes are currently described as direct targets of TBPH in Drosophila melanogaster. Both HDAC6 and futsch mRNAs are bound by TBPH. In particular, TBPH silencing decreases HDAC6 mRNA and, as a consequence, protein levels. On the other hand, TBPH silencing causes a drop only in futsch protein levels but not in mRNA expression, suggesting in this case an involvement of TDP-43 in either the mRNA transport or translations systems.

 At present, not many TBPH-interacting molecules have been identified that can possess a direct link with disease. Two exceptions are represented by the validated interaction between TBPH and the HDAC6 and *futsch* mRNAs (Figure 3).

 The histone deacetylase 6 (HDAC6) gene has been implicated in the pathogenesis of Alzheimer's disease and Parkinson's disease by linking together two protein degradation pathways (the ubiquitin proteasome system and autophagy) [66–69]. Recently, HDAC6 mRNA was identified as the first molecular target of TDP-43 conserved both in mammalian and invertebrates [9]. The levels of HDAC6 mRNA and protein decreased upon TDP-43 silencing and this effect was associated with accumulation of acetyl-tubulin, the major HDAC6 substrate [9]. It should also be noted that follow-up studies found that TDP-43 and FUS/TLS form a complex with HDAC6 [61]. These findings, therefore, suggested the possibility that alterations of TDP-43 or FUS/TLS might deregulate HDAC6 functions and this event could be crucial for ALS pathogenesis.

 In parallel, starting from the observation by different research groups that the loss of TBPH in Drosophila alters the morphological organization of the NMJ [6, 13, 17], further molecular characterization of the first model of TBPH-ko Drosophilas focused on possible alterations of elements important for the cytoskeletal organization at synaptic level. Among all the tested proteins, a consistent reduction in the protein levels of *futsch* was observed in the heads of TBPH -/- flies [19]. The Futsch protein is a homolog of human MAP1B necessary for proper axonal and dendritic growth [70]. In this study, it was shown that the TBPH protein directly interacts with *futsch* mRNA without affecting its stability or splicing profile. However, since futsch protein levels were decreased in TBPH -/- animals, this observation suggested that TBPH might be somehow involved in the posttranscriptional regulation of *futsch* expression [19]. This observation provided additional insights into the physiological roles of TDP-43 and into the potential mechanisms underlying ALS and the neurodegenerative diseases associated with alteration of TDP-43.

## 8. Concluding Remarks

 Altogether, the studies with Drosophila models have allowed us to make several general observations.

1. The data gained so far from these models significatively overlap most of the observations made in human patients and in rodent models, so strengthening the usefulness of the fruit fly in investigating the role played by TDP-43 protein abnormalities in ALS and other neurodegenerative diseases.
2. Similarly to what is observed in rodent models, both overexpression and silencing of TDP-43/TBPH in Drosophila produce neurodegeneration. Beyond the particular attention that must be reserved to the expression levels of TDP-43 mutants, whose effect should be tested specifically for any model, these observations also suggest that events able to modify the expression of TDP-43 in humans might elicit neuronal dysfunctions even before the appearance of pathological TDP-43 inclusions.

While Drosophila models confirm that toxicity occurs regardless of inclusions, the relevance of the cytoplasmic accumulation for the pathology remains an open question. In flies, expression of mutants accumulating in the cytoplasm showed an increase in toxicity, but a NLS mutant TDP-43 expressed in C. elegans, although cytoplasmic, did not result in alteration of locomotion and this observation led to the suggestion that nucleus is the primary site of the TDP-43 toxicity [71].

1. Different Drosophila models support the hypothesis that TDP-43 toxicity depends also on retaining the ability to bind RNA targets [8, 11, 19]. This observation further strengthens the hypothesis that the “loss of function” within nuclei is crucial for development of the disorder.
2. The carboxy-terminal domain seems to have a prominent role in the pathogenesis of human diseases. In fact, most mutations found in patients lie within this domain (for a recent review see [28]). Intriguingly, although the C-terminal domain is quite different in human and fly, the splicing regulation by human and Drosophila TDP-43 requires its presence. In fact, both TDP-43 and TBPH mutants devoid of this region lack splicing-modifying capacity. Therefore, although the structural conservation of this region is low in human and flies, some functions potentially associated with neurodegeneration have certainly been conserved through evolution.
3. Several studies have addressed the pathogenic role of hyperphosphorylation on the formation of TDP-43 inclusions [28–34]. It should be noted that none of the potential phosphorylated sites within the C-terminal region is conserved in flies (Figure 1(b)). However, Drosophila along with cellular models were also created to investigate this aspect by expressing human TDP-43 carboxyl terminal fragment [16]. Contrary to the previous, this study has suggested that phosphorylation occurs after aggregation and that aggregation propensity of TDP-43 could be reduced by hyperphosphorylation. The discrepancy between these results and previous studies could be ascribed to the different systems used for the characterizations.
4. Age-dependent degeneration is an important feature of neurodegenerative diseases. The investigations aimed at understanding the relevance of aging in affecting TDP-43 expression in humans and its possible association with Alzheimer were not conclusive [72]. At present, some of the Drosophila models of TDP-43-pathologies described in this paper support the hypothesis that aging can impact on the neurodegeneration caused by this nuclear factor [11, 13, 17]. However, additional studies are required to understand how many observation made in these flies models regarding this particular issues can be generalized to humans.

For all these reasons, fruit flies remain an appealing system in which to continue the characterization of the pathogenesis of TDP-43 proteinopathies through the screening of modifier genes or the search for drugs useful for the prevention and cure of neurodegenerative diseases.

## Figures and Tables

**Table 1:** Flies models of TDP-43 proteinopathies. Elav-GAL4 and 1407-GAL4 are pan-neuronal drivers; GMR-Gal4 driver is eye specific. D42-Gal4 driver is specific for motor neurons; OK107-GAL4 driver is specific for mushroom body. MHC-GAL4 and 24B-GAL4 drivers are muscle specific; Repo-GAL4 driver is glia specific.

	Loss of function (null allele)	Gain of function/RNAi transgenes	Phenotypes
Feiguin et al. [6]	Generation of mutants carrying chromosomal deletions. Two excised lines (TBPH D23 and TBPH D142) show small 1.6 and 0.8 kb deletions, respectively (that partially removed part of TBPH regulatory and coding regions)	Generation of transgenic flies with TBPH and hTDP-43 add back in TBPH knockout backgrounds using the D42-GAL4 or elav-GAL4 drivers.	Homozygous TBPH-KO flies were viable after embryogenesis. Homozygous flies that got rid of the external cuticle presented dramatic locomotive defects with spastic, uncoordinated, movements, incapacity to fly or walk normally and reduced life span. In TBPH-KO larvae, the number of axonal branches and synaptic boutons inside the muscles were reduced in the presynaptic terminals. Loss of TDP-43 function alters the morphological organization of the NMJ.
		TBPH RNAi lines were obtained.	RNAi caused similar locomotive defects

Lu et al. [7]	Generation of null allele carrying nonsense mutation with a single nucleotide change (G>A introduced a stop codon at codon 367 in TBPH-43 Q367X)	Generation of hTDP-43WT and hTDP-43 Q331K or hTDP- 43 M337V UAS-hTDP-43 transgenic flies.	Homozygosity for TBPH-43Q367X was semi-lethal, with some mutant adult flies surviving to adulthood. The number of small terminal dendritic branches was increased by overexpression of TBPH-43. Their localization was concentrated near the cell body of ddaE neurons. Ectopic expression of Q331K and M337V mutant proteins promoted dendritic branching to a much lesser extent than wild-type hTDP-43.
		Expression of UAS-TBPH-43 RNAi (38377 or 38379, VDRC) driven by tubulin-Gal4	RNAi resulted in a similar lethal phenotype.

Voigt et al. [8]		Generation of synthetic mutants (TDP-43SM) and ALS/FTLD-linked TDP-43MS TBPH-43 transgenic UAS/Gal4 fly lines: TDP-43SM variants (WT; ΔNLS; F147L/F149L; CTF, lacking the N-terminal portion including RRM1). ALS/FTLD-linked TDP-43MS variants (A315T;G287S;A382T;N390D).	All TDP-43 variants but TDP-43CTF and TDP-43FFLL caused premature lethality. All TDP-43 variants caused reduction in life span. Dose dependency of TDP-43-mediated neurotoxicity (TDP-43WT and TDP-43NLS). Although still alive, 20d-old TDP-43_WT expressing flies appeared paralytic, hardly showed coordinated movement, and failed to climb. Loss of motor neurons caused by TDP-43WT > TDP-43A315T and TDP-43DNLS.

Fiesel et al. [9]	Generation of null allele where the entire CDS of the TBPH gene was deleted		TBPH -/- animals die as second-instar larvae. The ability of hTDP-43 to bind and regulate expression of the HDAC6 mRNA is conserved also by TBPH and dhdac6 in Drosophila.

Hanson et al. [10]		To test the consequences of the TDP-43/UBQLN interaction *in vivo*, transgenic Drosophila lines were created with cDNAs encoding either wild-type human TDP-43 or human UBQLN under UAS/GAL4 control. GMR-Gal4 driver was used to express human TDP-43 in the fly eye. D42-Gal4 driver was used to express TDP-43 exclusively in motor neurons.	Overexpression of TDP-43 is toxic in the fly eyes. TDP-43 toxicity is both dose dependent and age dependent. TDP-43 expression in motor neurons reduces life span. UBQLN increases TDP-43 toxicity in both Drosophila and mammalian systems.

Li et al. [11]		Expression of hTDP-43 in Drosophila eyes with GMR-Gal4 driver beginning at the third instar larva. Overexpressed hTDP-43: WT or mutant T202 (containing the carboxyl-terminal glycine-rich domain but lacking the amino-terminal RNA recognition motif).	Overexpression of TDP-43 is toxic in the fly eyes. Both overexpression and silencing of hTDP-43 in mushroom bodies causes axonal and neuronal loss. Expression of hTDP-43 in motor neurons causes formation of aggregates in cell bodies and axons, as well as axon swelling. hTDP-43-expression causes age-dependent reduction in flies motility.
		Expression of hTDP-43 in Mushroom Bodies with OK107-Gal4 driver.	
		Expression of hTDP-43 in a small subset of MNs at the adult stage with RN2-Gal4 driver	
		TBPH RNAi lines were obtained.	

Ritson et al. [12]		Generation of transgenic flies expressing human WT or mutant TDP-43 using the UAS/GAL4 system. Used NES-mutant TDP-43 (nuclear) and NLS-mutant TDP-43 (cytoplasmic). TDP-43 (WT or mutant) under control of the driver fkh-GAL4.	In the eye, expression of dVCP-wt caused a modest phenotypic change, whereas matched expression of the R152H and A229E mutants caused severe external rough eye phenotypes with necrotic patches and vacuolar degeneration. Generation of transgenic lines overexpressing TBPH resulted in a degenerative phenotype when targeted to the eye.
		Expression of the ALS-causing mutation M337V (leads to toxicity associated with cytoplasmic redistribution of TDP-43).	The ALS-causing mutation M337V expressed *in vivo* leads to toxicity associated with cytoplasmic redistribution of TDP-43.
		Flies transgenic for UAS-dVCP (WT or mutant), UAS- TBPH, and UAS-TDP-43 (WT or mutant) were generated	Coexpression of exogenous TBPH with dVCP R152H enhanced degeneration associated with mutant VCP and confirming the genetic interaction.

		TBPH RNAi lines were obtained.	
Estes et al. [13]		Transgenic lines were generated to express TBPH and hTDP-43 wild-types and A315T mutants. Gal4 drivers used included the GMR-Gal4 and the D42-Gal4.	Overexpression of WT- and A315T-TDP-43 is toxic for the retina of fly eyes. TBPH-wt is 100% lethal when overexpressed at higher levels (by raising the temperature at 29°C). Wild-type and mutant TDP-43 create axonal aggregates in the developing eyes. Motor neurons expressing TDP-43 variants exhibit morphological defects at the NMJ synapse. Locomotor activity, viability, and survival are impaired regardless of the presence of detectable TDP-43 cytoplasmic aggregates.
		TBPH RNAi lines were obtained.	TBPH-RNAi enhances the toxic effect of both wild-type and A315T hTDP-43 expression in motor neurons.

Guo W. et al. [14]		Expression of hTDP-43 (WT or A315T) with OK371-Gal4 driver in subsets of motor neurons.	Flies often failed to eclose and surviving flies were smaller than control flies. Neurotoxicity and motor neuron deficits with mutant TDP-43. Expression of either wild-type or A315T mutant TDP-43 caused axonal abnormalities. Motor neurons expressing wild-type or mutant TDP-43 showed axonal swelling. Expression of A315T caused frequent fly death before the 3rd-instar stage. Surviving flies showed a marked axonal loss. In the remaining axons we detected severe damage, including axon swelling, axon thinning, and defects in axonal integrity. Expression of A315T (compared to wild-type) TDP-43 caused higher neuronal loss.

Lanson et al. [15]		The genetic interaction between human FUS/TLS and TDP-43 was tested by using transgenic TDP-43 flies described by Ritson et al.	Ectopic expression of mutant FUS/TLS leads to neurodegeneration in Drosophila. Genetic interaction between human FUS/TLS and TDP-43 tested by using transgenic TDP-43 flies described by Ritson et al. Eye expression of FUS WT or TDP-43 WT alone did not cause significant degeneration.
			Coexpression of both proteins caused moderate eye degeneration. Co-expression of FUS WT with M337V-TDP-43 led to severe eye degeneration. Co-expression of R521H FUS with TDP-43 WT synergistically enhanced the degeneration.

Li et al. [16]		GMR-Gal4 and elav-Gal4 were used to drive expression of wt (fTDP) and three truncated forms of TDP with N-terminal 104 (ND104), 207 (ND207), and 251 (ND251) amino acids deleted.	ND104, ND207, and ND251 were concentrated and formed aggregates in cytoplasm because of the lack of nuclear localization signal (NLS). The pattern of insoluble ND251 highlighted a shorter fragment and high molecular weight, similar to what is observed in TDP-ALS/FTLD-U inclusions.

Lin et al. [17]		Different GAL4 drivers were used to induce tissue-specific TBPH knockdown (Elav4;OK107; D42; MHC).	Pan-neuronal TBPH knockdown caused reduction in the moving abilities of larvae.
			Motor neurons TBPH overexpression reduced fly locomotor activities, along with a decrease of the number of boutons and axon branches at NMJ. TBPH overexpression in the mushroom bodies caused smaller axonal lobes and learning deficiency. Mushroom body-specific TBPH-knockdown did not affect the structure of the mushroom bodies, but caused modest reduction in the learning ability. TBPH-overexpression caused the formation of cytosolic TBPH aggregates.

Miguel et al. [18]		Different GAL4 drivers were used to induce tissue-specific TBPH-knockdown (Elav4; Repo; 24B; GMR).	Expression of TDP-43 wild-type is toxic for Drosophila eyes, muscles, and glia.
			Both nuclear and cytoplasmic TDP-43 accumulations are toxic for neurons (regardless of the formation of inclusions). Expression of human TDP-43 in adult flies neurons results in abnormally phosphorylation of the disease-specific Ser409/Ser410 residues and presence of high molecular weight forms in flies.

Godena et al. [19]		The previously generated TBPH-ko fly lines (Feiguin, 2009) were used to characterize the pathological consequences of TBPH-43 altered function in the Drosophila neuromuscular junctions during larval development.	TDP-43 is necessary for presynaptic microtubule organization: TBPH was found to modulate the expression of futsch, a neuron-specific microtubule binding protein ortholog of the human MAP1B important for maintaining the microtubule integrity during neuromuscular junctions expansion.

**Table 2:** Ectopic expression of TDP-43 mutants.

Human TDP-43 mutations		
Q331K	Eye	[7]
M337V	Eye	[7]
M337V	Eye	[12]
M337V	Eye	[15]
A315T	Eye	[13]
G287S	Pan-neural, ubiquitary	[8]
A315T	Pan-neural, ubiquitary, eye	[8]
G348C	Pan-neural, ubiquitary	[8]
A382T	Pan-neural, ubiquitary	[8]
N390D	Pan-neural, ubiquitary	[8]

Other mutants		
TDP-43 T202	Eye	[11]
TDP-43 NES-mut	Eye	[12]
TDP-43 NLS-mut	Eye	[12]
TDP-43 ΔNLS	Eye, pan-neural, ubiquitary	[8]
TDP-43 FFLL	Eye, pan-neural, ubiquitary	[8]
TDP-43 CTF	Pan-neural, ubiquitary	[8]
TBPH F/L 150–152	Pan-neural	[19]
hTDP-43mutNES	Eye, muscle, glia, pan-neural	[18]
hTDP-43mutNLS	Eye, muscle, glia, pan-neural	[18]

TDP-43 T202 contained only the C-terminal domain (the N-terminal RRM motifs are lacking). TDP-43 ΔNLS lacked a functional nuclear localization signal. TDP-43 FFLL contained two missense mutations (F147L/F149L) into the first RNA recognition motif (RRM1) that abolished TDP-43 RNA-binding function [35]. TBPH F/L 150-152 contained point mutations of the two Phe > Leu in RNP-2 of RRM1 corresponding to human (F147L and F149L). TDP-43 CTF consisted in the C-terminal fragment (206–414) similar to those found in cytosolic aggregates of ALS/FTLD patients. TDP-43 NLS-mut and hTDP-43mutNLS were mutants in the nuclear localization sequence (NLS). TDP-43 NES-mut and hTDP-43mutNES were mutants in the nuclear export sequence (NES).

## Abbreviations

ALS: Amyotrophic lateral sclerosis
FTLD: Frontotemporal lobar degeneration
NMJ: Neuromuscular juction
IBMPFD: Inclusion body myopathy associated with Paget's disease of bone and/or frontotemporal dementia
UBQLN: Ubiquilin 1
FUS/TLS: Fused in sarcoma/translocated-in-liposarcoma
HDAC6: histone deacetylase 6
TDP-43: TAR-DNA binding Protein-43
TBPH: TAR-DNA binding Protein Homologue.
